# Exploiting ROS and metabolic differences to kill cisplatin resistant lung cancer

**DOI:** 10.18632/oncotarget.17568

**Published:** 2017-05-02

**Authors:** Medhi Wangpaichitr, Chunjing Wu, Ying Ying Li, Dan J.M. Nguyen, Hande Kandemir, Sumedh Shah, Shumei Chen, Lynn G. Feun, Jeffrey S. Prince, Macus T. Kuo, Niramol Savaraj

**Affiliations:** ^1^ Miami VA Healthcare System, Research Service, Miami, FL, USA; ^2^ Department of Surgery, Cardiothoracic Surgery, University of Miami, Miami, FL, USA; ^3^ Department of Medicine, Hematology/Oncology, University of Miami, Miami, FL, USA; ^4^ Department of Biology, University of Miami, Miami, FL, USA; ^5^ School of Medicine, Koc University, Istanbul, Turkey; ^6^ Department of Neurosurgery, Taipei Medical University-Wan Fang Hospital, Taipei, Taiwan; ^7^ Department of Translational Molecular Pathology, University of Texas MD Anderson Cancer Center, Houston, TX, USA

**Keywords:** lung cancer, reactive oxygen species, oxidative metabolism, riluzole, resistance

## Abstract

Cisplatin resistance remains a major problem in the treatment of lung cancer. We have discovered that cisplatin resistant (CR) lung cancer cells, regardless of the signaling pathway status, share the common parameter which is an increase in reactive oxygen species (ROS) and undergo metabolic reprogramming. CR cells were no longer addicted to the glycolytic pathway, but rather relied on oxidative metabolism. They took up twice as much glutamine and were highly sensitive to glutamine deprivation. Glutamine is hydrolyzed to glutamate for glutathione synthesis, an essential factor to abrogate high ROS via xCT antiporter. Thus, blocking glutamate flux using riluzole (an amyotropic lateral sclerosis approved drug) can selectively kill CR cells *in vitro* and *in vivo*. However, we discovered here that glutathione suppression is not the primary pathway in eradicating the CR cells. Riluzole can lead to further decrease in NAD^+^ (nicotinamide adenine dinucleotide) and lactate dehydrogenase-A (LDHA) expressions which in turn further heightened oxidative stress in CR cells. LDHA knocked-down cells became hypersensitive to riluzole treatments and possessed increased levels of ROS. Addition of NAD^+^ re-stabilized LDHA and reversed riluzole induced cell death. Thus far, no drugs are available which could overcome cisplatin resistance or kill cisplatin resistant cells. CR cells possess high levels of ROS and undergo metabolic reprogramming. These metabolic adaptations can be exploited and targeted by riluzole. Riluzole may serve as a dual-targeting agent by suppression LDHA and blocking xCT antiporter. Repurposing of riluzole should be considered for future treatment of cisplatin resistant lung cancer patients.

## INTRODUCTION

Drug resistance is a major obstacle to cancer chemotherapy. Despite early positive response to platinum-based chemotherapy, the majority of small-cell and non-small cell lung cancer (SCLC & NSCLC) develop resistance. We have discovered that elevated reactive oxygen species (ROS) are found in all cisplatin resistant (CR) cell lines including those derived from patients who failed cisplatin. Further increasing ROS using a ROS inducing agent can selectively kill CR cells [1]. We also have presented the evidence that decreased thioredoxin-1 (TRX1) expression in CR lung cancer cells is one important mediator of ROS and reprograms lung cancer cells to become more reliant on oxidative metabolism (OXMET) [1]. Thus, unlike the parental cells or cells from chemo-naïve patients, these resistant cells are not utilizing glycolysis as their main source of energy. It is known that tumor cells metabolize glucose to lactate even when oxygen is abundant (Warburg effect) [2–5]. This is not due to defective mitochondrial respiration but rather due to up-regulation of glycolytic enzymes and glucose transporters [6–8]. In fact, increased glucose uptake is one of the hallmarks for malignant transformation [9, 10].

While decreased TRX1 may be a primary factor which contributes to increasing ROS and metabolic switch in CR cells, there are other factors involved since restoration of TRX1 cannot completely reverse cisplatin resistance. In this manuscript, we study other mechanisms involved and methods to circumvent this form of resistance. We have found that CR cells expressed significantly lower levels of hexokinase-2 (HK2) and lactic dehydrogenase-A (LDHA) which further indicated that CR cells do not depend primarily on glycolysis. Moreover, studies have shown that reduction in LDHA expression in cancer cells either by genetic knock down (shRNA) or inhibitor (FX11) resulted in the shift to oxidative phosphorylation (OXPHOS) and increased intracellular ROS [11, 12]. These studies have elucidated that LDHA mediated inhibition of mitochondria respiration and enhanced glycolysis in cancer cells, thus LDHA could potentially be considered as a therapeutic target. Since our previous data indicated that CR cells have very low lactic acid production, we plan to determine the role of LDHA and its important co-factor NAD^+^ (nicotinamide adenine dinucleotide) in CR cells.

Our findings also revealed that unlike parental cells, CR cells were not sensitive to glucose withdrawal, but were exclusively sensitive to glutamine deprivation which further supported our hypothesis that CR cell rely on OXMET as their main energy source. Thus, it is possible that interfering with glutamine and glutamate flux can be used to target CR cells. It is known that riluzole, an FDA-approved orally available drug for the treatment of amyotropic lateral sclerosis (ALS) [13], interferes with glutamate flux and blocks metabotropic glutamate receptors (GRM) signaling. This drug was shown to block proliferation of melanoma cell lines expressing GRM *in vitro* and *in vivo* while inhibition with a dominant negative construct resulted in apoptosis [14]. A phase-0 trial of riluzole in melanoma also showed good response [15].

In this study, we exploited ROS and metabolic alterations, as well as explored the possible antitumor effect of riluzole in CR cells.

## RESULTS

### Cisplatin resistant (CR) lung cancer cells were no longer addicted to glucose

**Figure 1:**
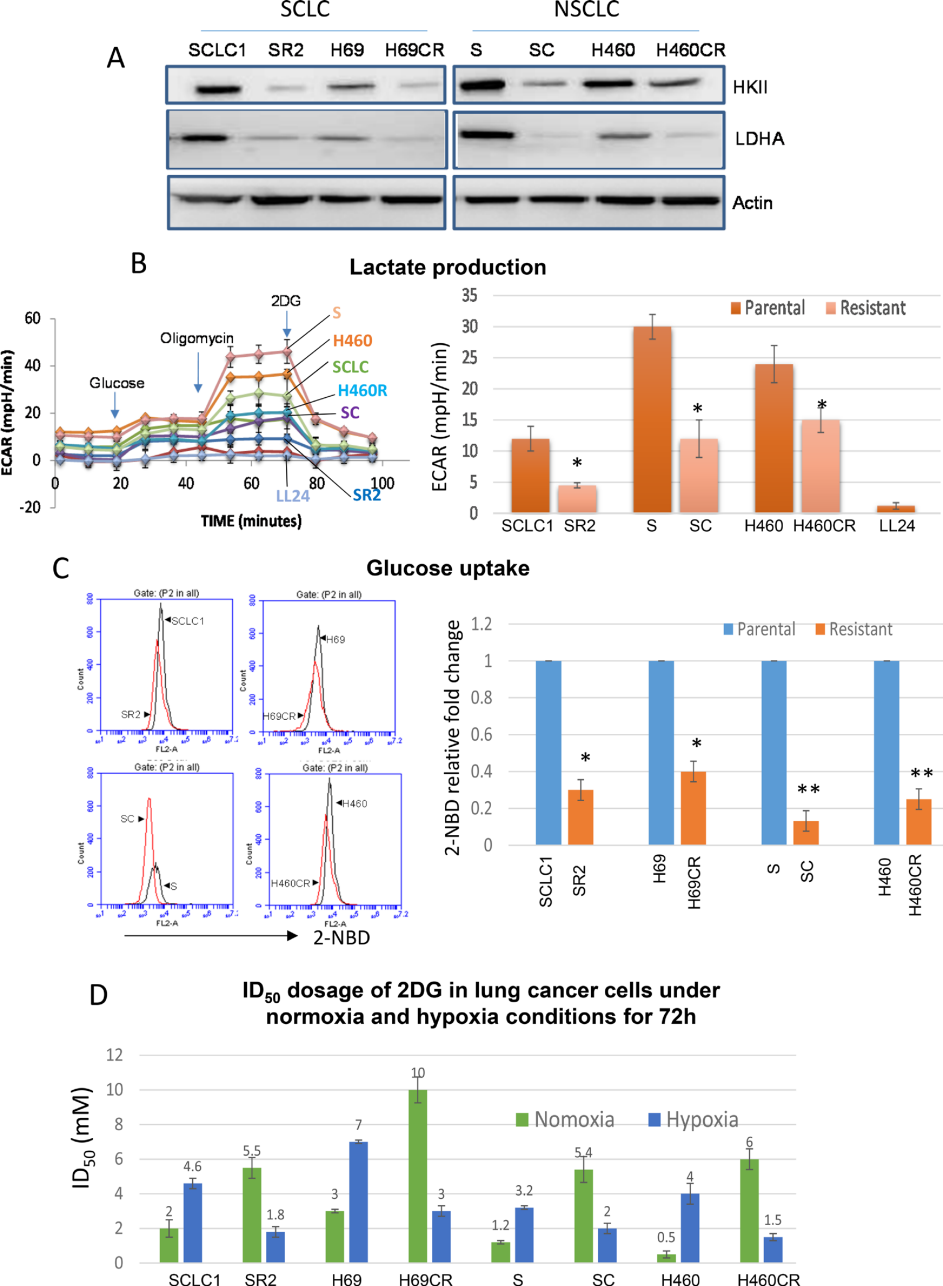
CR lung cancer cells do not mainly rely on glycolysis (**A**) Immunoblot of lung cancer cell lines showed that resistant variants expressed lower levels of HK2 and LDHA. Actin was used as a loading control. (**B**) Lactate production measured by Seahorse XF^e^24 extracellular flux analyzer indicated that CR cells produced significantly lower levels of lactic acid (*P < 0.015). LL24 is normal lung fibroblast. Note that H69 vs. H69CR cannot be used in this assay due to the floating aggregate nature of the cells which interfered with accurate measurement. Left panel: the schematic presentation of the experimental workflow. Right panel: extrapolated data from Seahorse report generator. Supplementary Figure 1A showed the schematic of glycolytic function test. (**C**) Flow cytometer analysis showed that parental cells (black peak) uptake higher levels of fluorescent glucose analog (2-NBD) when compared to CR cells (red peak). Right panel illustrated 2-NBD fold change with parental cells were set at 1 (*p < 0.05, **p < 0.02). (**D**) Growth inhibitory dosage (ID_50_) of 2-DG for 72 h showed that CR were resistant to 2-DG in normoxia, but became sensitive when placed under hypoxia (Mean SD of three experiments).

We have previously shown that increased secretion of the antioxidant thioredoxin-1 (TRX1) resulted in lowered intracellular TRX1, and contributed to higher ROS in cisplatin resistant (CR) tumors (Supplementary Figure 1). Consequently, alterations in metabolic pathway were found in CR cells. To verify this, we have assayed the key enzymes in the glycolytic pathway. Our results showed that all CR cells expressed lower levels of HK2 and LDHA proteins (Figure 1A). Using Seahorse XF^e^24 Extracellular flux analyzers, we assayed for lactate production in response to adding glucose, oligomycin, and 2DG (Figure 1B, left panel), our results indicated that CR produced significantly less lactate (Figure 1B, right panel). To further support that CR cells are less addicted to glucose, we demonstrated that CR took up less fluorescent glucose analog (2-NBD) by flow cytometry when compared to parental cell counterparts. All CR cells' peaks were shifted to the left as depicted in Figure 1C. As a result, CR cells were more resistant to glycolytic inhibitor, 2-deoxy-glucose (2DG), with an average of 2–5 fold higher under normoxia (Figure 1D). To further confirm that CR cells were less capable of utilizing glycolysis, we performed growth inhibitory assay under the hypoxic condition (0.5%O_2_). Under this condition, tumor cells which utilized glycolysis survived; however, CR cells could not proliferate nor survive under this condition and became more sensitive to glycolytic inhibitor (Figure 1D). Taken together, our findings strongly suggested that CR cells were no longer addicted to glucose.

### Higher mitochondrial activities were found in CR cells

**Figure 2:**
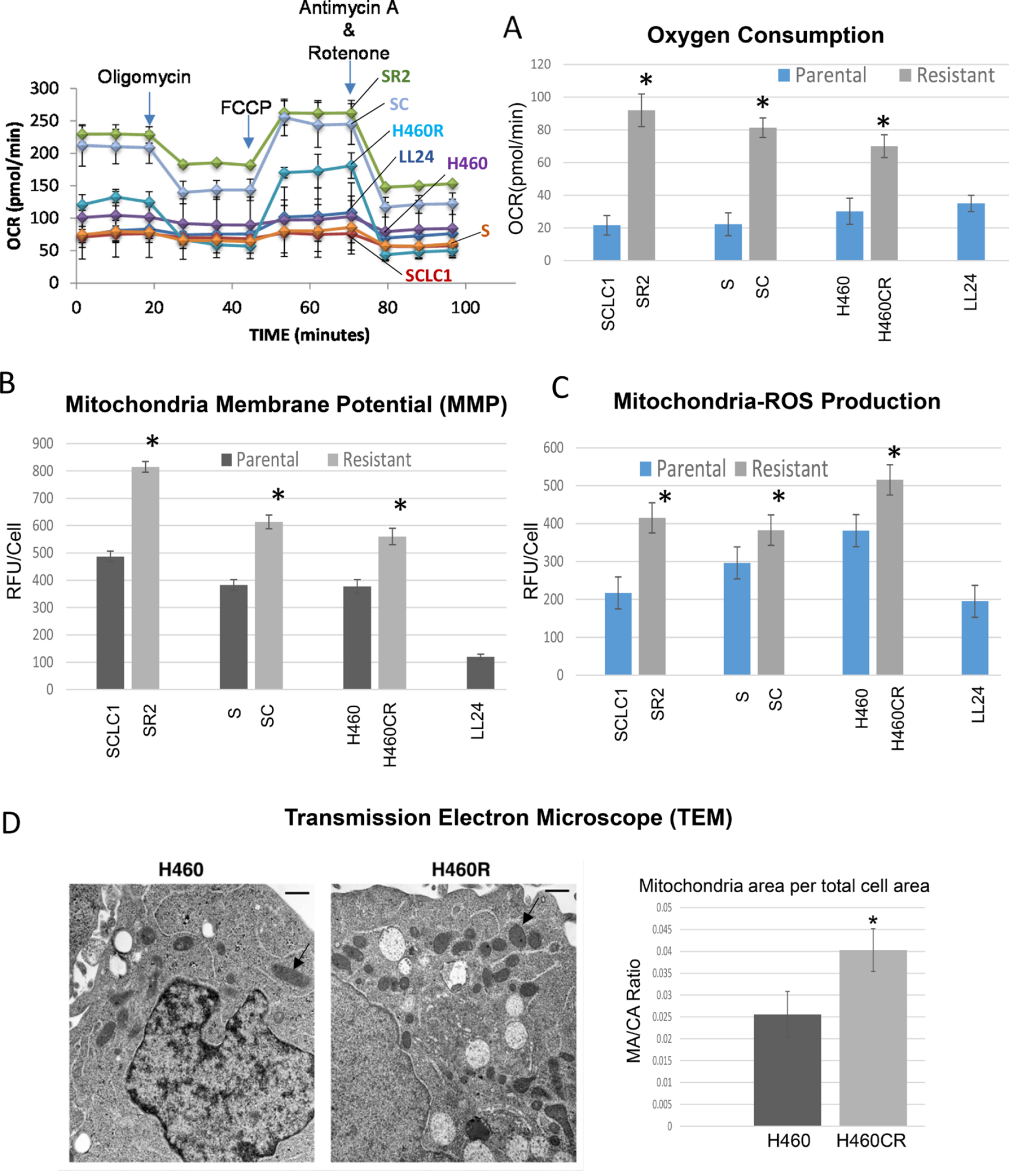
CR lung cancer cells relied on oxidative metabolism (**A**) Parental and CR cells were assayed for baseline oxygen consumption using Seahorse XF^e^24 extracellular flux analyzer. The rate of oxygen consumption (OCR) was significantly higher in CR than parental cells (*P < 0.003). LL24 was used as control. Left panel: the schematic presentation of the experimental workflow. Right panel: extrapolated data from Seahorse report generator. Supplementary Figure 2A showed the schematic of mitochondrial respiration test. (**B**) Analysis of MMP in Parental vs. CR cells, using 50 nM of TMRE; CR cells possess significant higher levels of MMP (Mean SD of three experiments; *average *p <* 0.03). (**C**) Mitochondria-ROS analysis detected by MitoSOX probe indicates that CR cells expressed higher basal levels of mitochondria-ROS. Bar graph represents the relative fluorescent units/cell via fluorometer plate reader; (Mean SD of three experiments; average*p < 0.05). (**D**) TEM image of H460 vs H460CR; Arrow indicates mitochondrial organelle. Right panel: Quantitative analysis of electron micrographs show that H460CR possess significant higher amounts of mitochondria per the total cell area (*P = 0.0006).

Since CR cells were less addicted to glycolytic pathway, they must utilize mitochondria for biogenesis to catabolize alternative carbon skeleton source. To confirm this, we first compared oxygen consumption using Seahorse flux analyzer. In response to adding glucose, oligomycin, FCCP, and rotenone (Figure 2A left panel.), CR cells consumed significantly higher rates of oxygen (Figure 2A right panel), and thus had higher levels of ATP production when compared to their parental cells counterparts (Supplementary Figure 2, *p <* 0.01). CR cells also have increased mitochondrial membrane potential (MMP) as detected by TMRE (Figure 2B). To determine whether active mitochondria may lead to increased mitochondria-ROS production, we assayed for ROS levels in the cell line pairs using MitoSOX. As shown in Figure 2C, all CR cell lines tested indeed have higher basal levels of mitochondria-ROS. Together, our data suggested that CR cells utilized more OXPHOS and conceivably possessed higher mitochondria per cell when compared to their parental cell counterparts. To further verify this, we assessed mitochondrial alterations through the transmission electron microscope (TEM). CR cells possessed significantly higher number of mitochondria per total cell area when compared with parental (*p* = 0.0006) (Figure 2D). Overall, our data clearly indicated that CR cells utilized OXMET and do not depend primarily on glycolysis.

### Glutamine and glutamate flux greatly impact cell viable in CR cells

**Figure 3:**
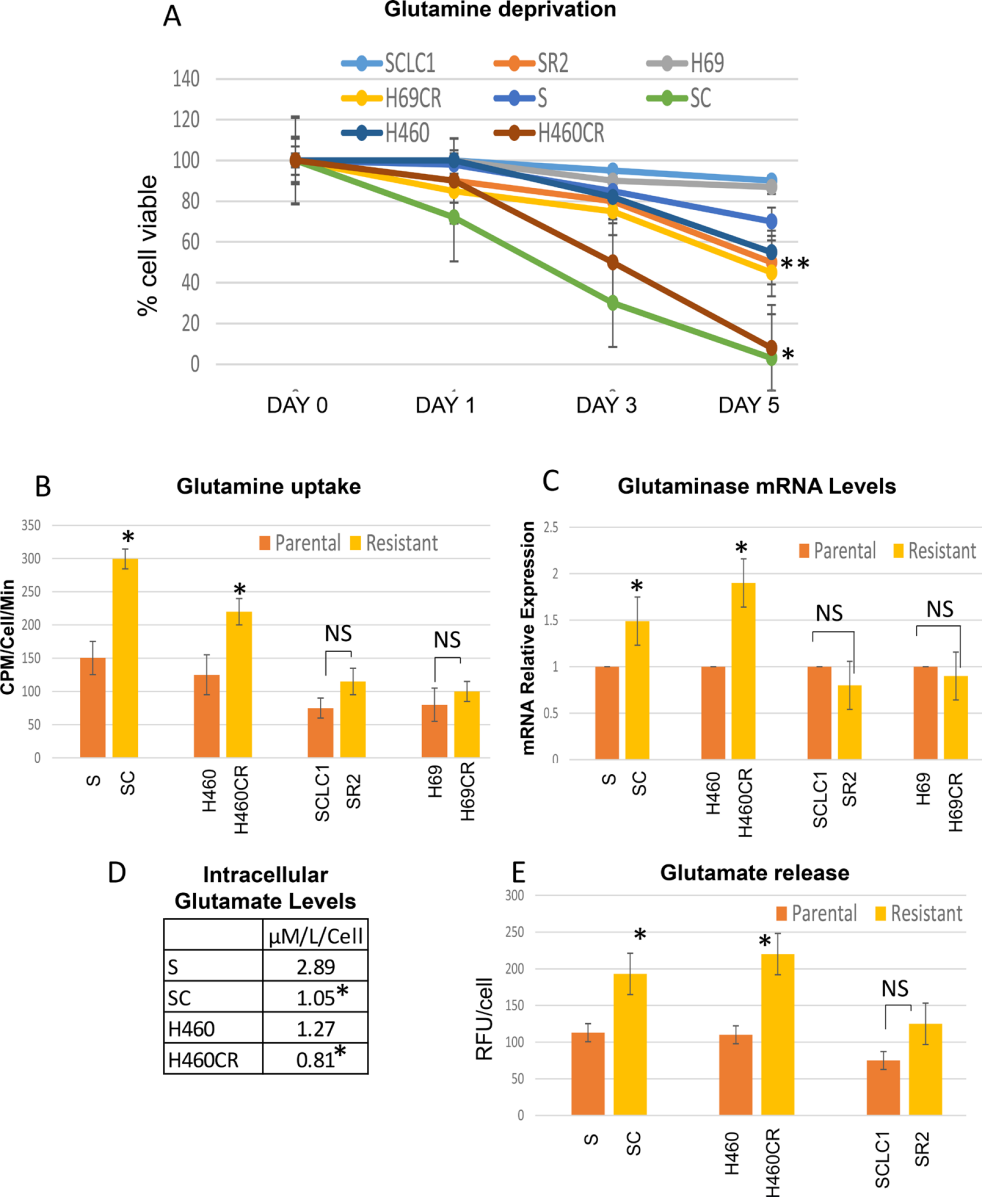
CR lung cancer cell lines depended more on glutamine (**A**) Effect of glutamine withdrawal on the viability of lung cancer cells. NSCLC-CR cells cannot survive without glutamine after 3 days (Mean SD of three experiments; average *p < 0.01, average **p < 0.05). (**B**) Glutamine uptake was determined in parental vs CR cells using L-[G-^3^H] glutamine. CR cells uptake higher rate of glutamine (*P < 0.035; NS = not significant). (**C**) Relative mRNA levels of glutaminase; Total RNAs extracted from these cells were reverse-transcribed and subsequently used as template for real-time quantitative PCR. Actin was used as internal control. The results shown in the graph were calculated with the ΔΔCt method by setting the glutaminase mRNA level of parental cells as 1 (*p < 0.03*;* NS = not significant). (**D**) CR cells possessed lower concentration of intracellular glutamate levels (*P < 0.05; parental vs CR). (**E**) Glutamate secretion assay; CR cells released significantly higher amount of glutamate (Mean SD of three experiments; *P < 0.02, NS = not significant).

To determine that CR cells indeed rely more on OXMET and not glucose, we investigated whether glutaminolysis is essential for CR cells to survive. We first compared cell viability of parental vs. CR cells in glutamine-free media. As shown in Figure 3A, glutamine withdrawals were able to inhibit growth and have a detrimental effect on all CR cell lines. The switch of glutamine from being a nonessential amino acid to an essential amino acid in CR cells was not an artifact of *in vitro* culture since glutamine deprivation only had the profound effect toward CR cells, but not in parental cells.

Moreover, CR cells took up higher amounts of glutamine especially in non-small cell lung cancer (NSCLC) which took up twice as much L-[G-^3^H] glutamine when compared to its parental counterpart (Figure 3B). Using qRTPCR to assay glutaminase (GLS), an enzyme that catalyzes the hydrolysis of glutamine to glutamate, we have found that the levels were higher in NSCLC but not in small cell lung cancer (SCLC) cells that were resistant to cisplatin (Figure 3C). We then investigated whether there were any differential sensitivity to GLS inhibitor (BPTES) seen in these CR cells compared to parental cells. Interestingly, our data showed that CR cells were slightly but not significantly sensitive to BPTES (Supplementary Figure 3). We then assayed for intracellular glutamate levels using amino acid analyzer and found that CR cells do not possess higher levels of glutamate inside the cells (Figure 3D). Conversely, we discovered that glutamate secretions were higher in CR cells' media and much higher in cisplatin resistant non-small cell lung cancer (NSCLC-CR) compared to cisplatin resistant small cell lung cancer (SCLC-CR) (Figure 3E).

Here, we reported the dynamics of glutamine and glutamate flux in CR cells. Higher glutamine uptake rates in CR cells also lead to increased levels of glutamate release which may possibly be metabolically exploited. However, in SCLC, it appeared that the flux of glutamine uptake as well as glutamate release were not significant when compared to NSCLC. Only marginally increased in glutamine/glutamate were observed. In addition, the levels of glutaminase levels were also lower in SCLC-CR compared to parental cells. Overall, it appeared that the glutamine/glutamate axis may not be a major amino acids utilizing source in SCLC. We therefore will focus on NSCLC cells herein.

### Exploiting glutamate efflux in cisplatin resistant non-small cell lung cancer (NSCLC-CR) cells using riluzole

**Figure 4:**
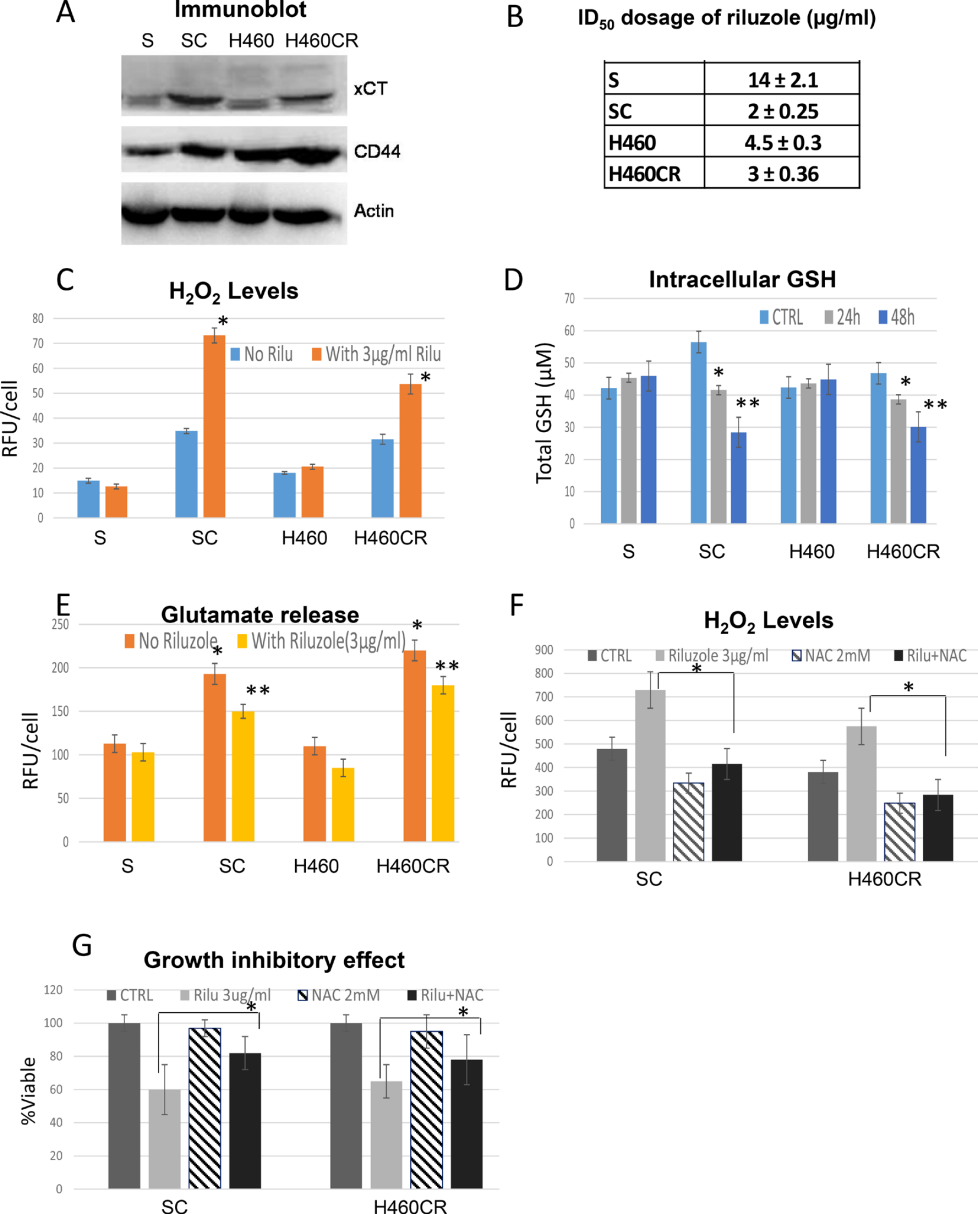
Exploiting glutamate efflux using riluzole (**A**) Immunoblot of lung cancer cell lines showed that resistant variants expressed higher levels of xCT and CD44. (**B**) Growth inhibitory dosage (ID_50_) of riluzole for 72 h showed that CR were hypersensitive to riluzole (Mean SD of three experiments). (**C**) H_2_O_2_ productions measured by APFB. CR cells possessed higher basal levels of ROS and were heightened when treated with riluzole (72 h). Bar graph represents the relative fluorescent units/cell via fluorometer plate reader (Mean SD of three experiments; average *P < 0.02). (**D**) Total cellular of GSH in lung cancer cell lines treated with 3μg/ml of riluzole for 24 and 48 h. Note: After treatment with riluzole, the levels of GSH were decreased in all CR cells (Mean SD of three experiments; average *P < 0.05, **P < 0.03). (**E**) CR cells released higher amount of glutamate (Mean SD of three experiments; *P < 0.02) and can be suppressed by riluzole treatment (48 h) (Mean SD of three experiments; average **p < 0.05). (**F**) H_2_O_2_ productions measured by fluorescence intensity. ROS were increased when treated with riluzole and can be suppressed by 2 mM of *N*-acetylcysteine (NAC) (48 h). Bar graph represents the relative fluorescent units/cell via fluorometer plate reader (Mean SD of three experiments; average *p < 0.02). (**G**) NAC was able to reverse the growth inhibitory effect of riluzole in CR cell lines (48 h) (Mean SD of three experiments; average *p < 0.05).

Since our data suggest that glutamine/glutamate was the major source of energy in NSCLC-CR, disrupting this pathway may result in metabolic catastrophe which in turn leads to cell death. To investigate this possibility, we studied the possible antitumor activity of the FDA approved drug (riluzole) which is a potent inhibitor of glutamate secretion [14, 16, 17]. Riluzole interferes with system xCT/cystine/glutamate pump which results in decreasing glutathione (GSH) levels inside the cells (see Figure Diagram). It has been shown that CD44 has a role in cellular response to oxidative stress [18, 19]. Here, we showed that CR cells, which possess higher basal levels of ROS, expressed higher levels of xCT and CD44 proteins (Figure 4A). Importantly, CR cells were very sensitive to riluzole treatment with significant lower ID_50_ dosage when compared to parental cell counterparts (Figure 4B and Supplementary Figure 4A; *p <* 0.03). ROS levels were increased significantly in CR cells after riluzole treatment (Figure 4C; *P <* 0.02). Simultaneously, total GSH concentrations were decreased within 24 h and further reduced at 48 h only in CR cells after treatment (Figure 4D). Since riluzole is known to inhibits glutamate efflux [20, 21], we assayed for glutamate release before and after riluzole treatment. Riluzole indeed was able to inhibit glutamate release in both parental and more significantly in CR cells (Figure 4E; *p* < 0.05).

**Figure Diagram:**
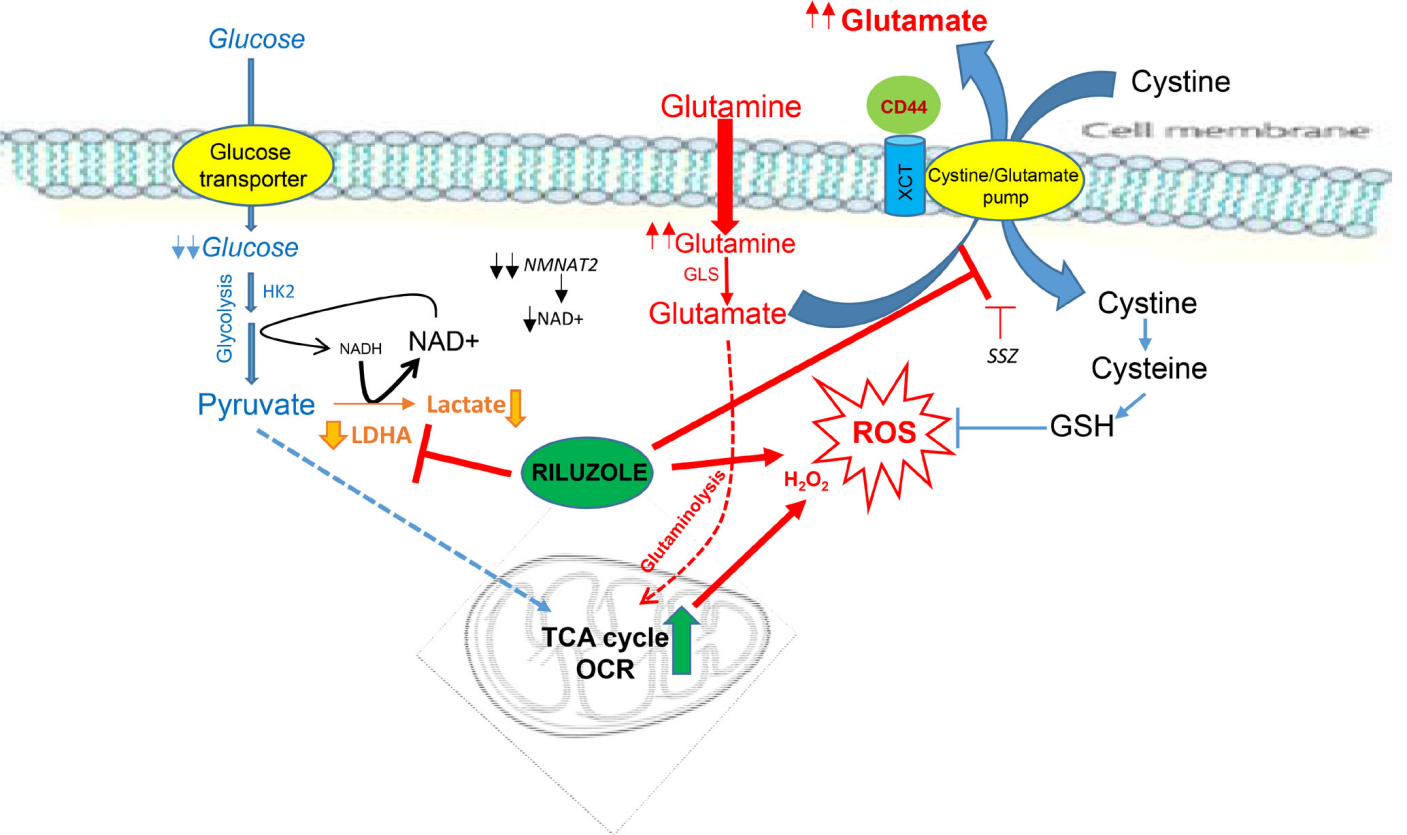
Cisplatin resistant (CR) cells consume more oxygen and possess higher basal levels of ROS CR cells are no longer depend on glycolysis but instead reliant on oxidative metabolism. Riluzole heightens ROS by further suppressing LDHA and NAD^+^. Riluzole also interferes with xCT antiporter which brings in cystine in-exchange for transporting out glutamate. Inhibit this pump results in reducing glutathione (GSH) levels inside the cells. (HK2: hexokinase-2, LDHA: lactate dehydrogenase-A, GLS: glutaminase, NMNAT2: nicotinamide mononucleotide adenylyltransferase-2, OCR: oxygen consumption rate, SSZ: sulfasalazine)

To determine whether cell death is associated with increased ROS, we exposed cells to N-acetyl-cysteine (NAC) in combination with riluzole. NAC is a well-known antioxidant and GSH precursor. Pretreatment with 2 mM of NAC significantly suppressed ROS and reversed riluzole cytotoxicity (Figure 4F; *P* < 0.02 and 4G; *p* < 0.05 respectively). We noted that 72 h combination treatment only partially suppressed ROS and reduced cell death. This may due to the short half-life of NAC activity. As a proof of concept, we treated Rho 0 (mitochondrial compromise cell) with riluzole. As expected, Rho 0 cells were very resistant to riluzole (Supplementary Figure 4B). Overall, these findings further support that riluzole induced CR cells death through mitochondria ROS.

In order to determine the functional relevance of xCT/cystine/glutamate anti-port pump, we treated CR cells with sulfasalazine (SSZ), a known inhibitor of system xc^−^ pump [22, 23]. This drug also has been used in combination with cisplatin in colon cancer [24]. However, in our system, CR cells were not sensitive to SSZ when compared to parental counterparts (Supplementary Figure 4C). ROS accumulations were observed in CR cells only when these CR cells were treated with supra-pharmacological dosage which cannot be achieved *in vivo*. Hence, our data suggested that cystine/glutamate anti-port is not the only mechanism involved. Riluzole must also affects other systems which together further amplified oxidative stress in CR cells.

Nonetheless, we showed here that CR cells required glutamine for OXMET and that glutamine-glutamate flux also played an important role in countering excessive ROS in these cells. Disrupting cystine/glutamate transporter system with riluzole resulted in higher intracellular ROS levels, which contributed to one of the mechanisms in killing CR cells.

### Treatment with riluzole results in decreasing LDHA expression

**Figure 5:**
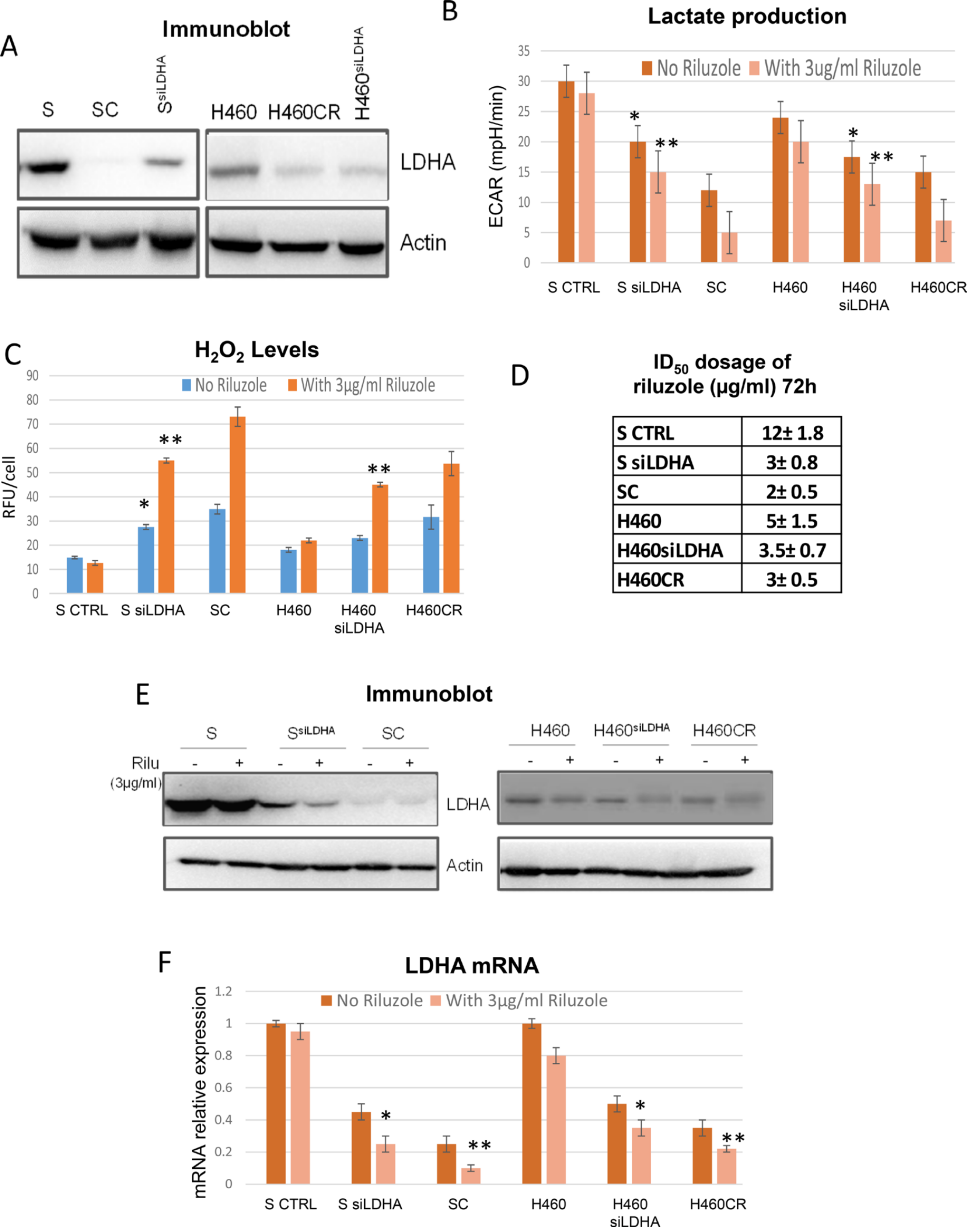
Riluzole affects LDHA levels (**A**) Immunoblot of LDHA; S and H460 contained scramble sequence while S^siLDHA^ and H460^siLDHA^ contained LDHA knocked-down. (**B**) Lactate production measured by Seahorse XF^e^24 extracellular flux analyzer indicated that lactate production was decreased after LDHA knocked-down (*p < 0.05) and further decreased with riluzole treatment (72 h) (**P < 0.01). (**C**) ROS production was increased after LDHA knocked-down (*p < 0.05) and further increased with riluzole treatment (72 h) (*p < 0.01). (**D**) Growth inhibitory dosage (ID_50_) of riluzole for 72 h showed that LDHA knocked-down were hypersensitive to riluzole. (**E**) Immunoblot of LDHA with and without riluzole treatment for 72 h. S and H460 contained scramble sequence while S^siLDHA^ and H460^siLDHA^ contained LDHA knocked-down. (**F**) Relative mRNA levels of LDHA showed that riluzole treatment (72 h) further suppressed LDHA expressions both in knocked down and CR cells (*P < 0.04 and **P < 0.02). Total RNAs extracted from these cells were reverse-transcribed and subsequently used as template for real-time quantitative PCR. Actin was used as internal control. The results shown in the graph were calculated with the ΔΔCt method by setting the LDHA mRNA level of parental cells as 1.

It has been shown that cells with higher ROS have lower LDHA expression [11]. Whether LDHA plays a key role in riluzole sensitivity seen in CR cells is not known, we then investigated this possibility by inhibiting LDHA expression in parental cell lines S and H460 using siRNA (Figure 5A). Lactate production was decreased in knockdown cells as anticipated and was further attenuated upon riluzole treatment when compared with scrambled control (Figure 5B). Higher basal levels of H_2_O_2_ were obtained from S^siLDHA^ and H460^siLDHA^ and increased levels in ROS were observed when treated with riluzole (Figure 5C). Likewise, reduction of LDHA expression by siRNA in parental cells also resulted in increased oxygen consumption and was further augmented when exposed to riluzole (Supplementary Figure 5). We then evaluated the viability of these cells under riluzole treatment. Both siLDHA clones were sensitive to riluzole, particularly S^siLDHA^ which showed 4-fold higher sensitivity (Figure 5D). Importantly, treatment with riluzole also led to further decrease in LDHA expression at both protein (Figure 5E) and mRNA levels (Figure 5F). To further support this notion, we treated SR2 cells which do not mainly depend on glutamine but possessed lower level of LDHA with riluzole. Indeed, SR2 were also sensitive to riluzole treatment (Supplementary Figure 4A). Here, our data indicated that LDHA suppression may be the main target of riluzole induced cell death in CR cells.

### Riluzole suppresses NAD^+^ and decreases LDHA levels

**Figure 6:**
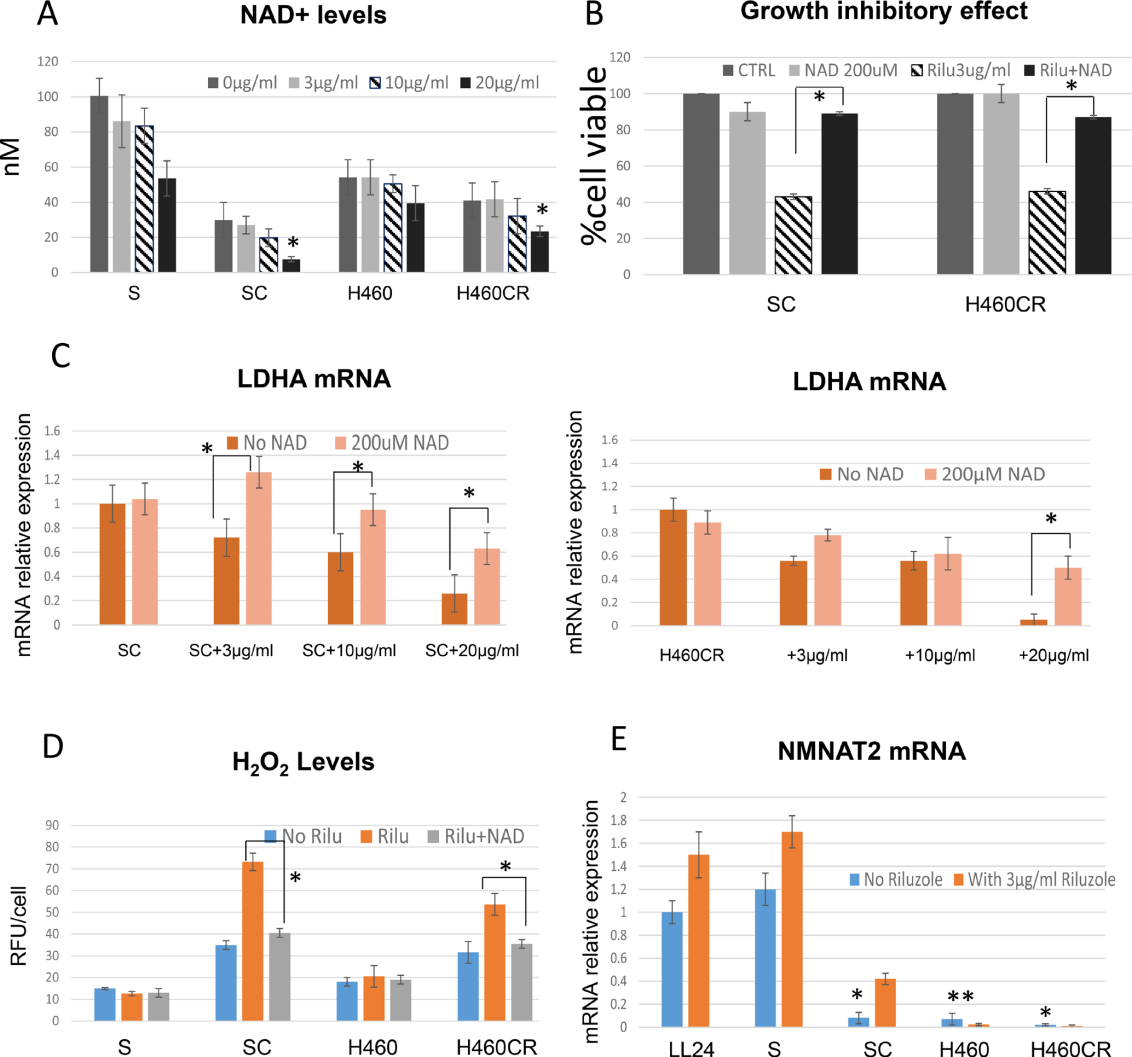
Riluzole also affects NAD^+^ levels (**A**) Total cellular NAD^+^ concentrations were detected in parental vs. CR cell lines. CR possessed lower basal levels of NAD^+^ and can be further reduced by addition of riluzole (1 h treatment) (*P < 0.02). (**B**) Growth inhibitory effect (72 h); Addition of 200 μM of NAD^+^ can reverse cytotoxic effect from riluzole (Mean SD of three experiments, average *p < 0.05). (**C**) Relative mRNA levels of LDHA; Treatment of NAD^+^ can re-stabilize LDHA expressions after riluzole (72 h) (*p < 0.03). Total RNAs extracted from these cells were reverse-transcribed and subsequently used as template for real-time quantitative PCR. Actin was used as internal control. The results shown in the graph were calculated with the ΔΔCt method by setting the LDHA mRNA level of control cells (SC or H460CR) as 1. (**D**) ROS production were increased with riluzole treatment, but suppressed when exposed to NAD^+^ (72 h) (*p < 0.02). (**E**) Relative mRNA levels of NMNAT2; NMNAT2 were significantly decreased in CR cells (72 h) (*p < 0.001). Note that H460 which do not have high LDHA levels also expressed significantly low level of NMNAT2 (**p < 0.004). Total RNAs extracted from these cells were reverse-transcribed and subsequently used as template for real-time quantitative PCR. Actin was used as internal control. The results shown in the graph were calculated with the ΔΔCt method by setting the NMNAT2 mRNA level of control cells (LL24) as 1.

Since NAD^+^ is a co-factor of LDHA, we assayed for NAD^+^ levels in parental vs. CR cells to further determine the important relationship between NAD^+^ levels and LDHA expression. As depicted in Figure 6A, SC cell line which expressed lower levels of LDHA also possessed significantly lower levels of intracellular NAD^+^. However, we also noted that the NAD^+^ levels in H460CR is only slight lower than H460. This is most likely due to the fact that the NAD^+^ levels were already low in parental cells (H460). Nevertheless, our data consistently indicated that lower NAD^+^ levels were correlated with riluzole sensitivity. Both SC and H460CR NAD^+^ levels were further suppressed upon riluzole treatment and increased riluzole concentrations resulted in greater suppression of NAD^+^ levels. To assess that NAD^+^ played a role in protecting cell against riluzole, we treated CR cells in combination with 200 μM NAD^+^. The addition of supplemental NAD^+^ reversed riluzole cytotoxicity almost completely (Figure 6B). We then assayed for LDHA expression to further determine the relationship between NAD^+^ and LDHA after riluzole treatment. LDHA expression was significantly decreased upon riluzole treatments, but can be reversed by addition of NAD^+^ (Figure 6C). Reduced ROS accumulations were also observed with combination treatments of NAD^+^ with riluzole (Figure 6D).

To further explore why the levels of NAD^+^ correlated with LDHA and riluzole sensitivity, we assayed for NMNAT2 (nicotinamide mononucleotide adenylyl transferase-2), a cytosolic enzyme that catalyzes the production of NAD^+^ from nicotinamide mononucleotide (NMN). NMNAT2 represents the final step in the biosynthesis of NAD^+^ in both *de novo* and salvage pathways. Consistent with LDHA expressions, CR cells expressed significantly lower levels of NMNAT2 (Figure 6E). It is also evident that NMNAT2 expressions were increased upon riluzole treatment in both parental and CR cells to compensate for NAD^+^ reduction. Nevertheless, increased NMNAT2 is not sufficient to escalate enough NAD^+^ biogenesis to overcome the loss of LDHA and NAD^+^. Interestingly, there were reports that suggested NAD^+^ can stimulate PKA activity and consequently stabilize LDHA mRNA [25, 26]. Thus, these works further supported our findings that decreased NAD^+^ levels by riluzole can impact LDHA protein integrity. This is an important issue that needs to be addressed. We plan to investigate these complex relationships in the future.

### GRMs (metabotropic glutamate receptors) are not involved in antitumor activity of riluzole

Since riluzole inhibits glutamate efflux [20, 21], and hence may affect metabotropic glutamate receptor (GRM) and its signaling pathway as one of the mechanisms in inhibiting cell growth. To further determine the relationship between glutamate signaling and riluzole sensitivities, we assayed for GRM1 expressions. The levels of GRM1 did not correlate with riluzole cytotoxicity. We then assayed for other variants of glutamate receptors (GRM1 to GRAM5) at the mRNA levels. Again, we found no correlation between GRM expressions with riluzole sensitivity (Supplementary Figure 6). Using flow cytometry to quantify functional GRM1 that was localized at the cell surface also revealed no correlation. Thus, our data did not support that GRM signaling participates in riluzole cytotoxicity seen in CR cell. This is also consistent with a recent report that silencing and overexpression of GRMS had no effect on cell sensitivity to riluzole [27].

### Antitumor activity of riluzole in CR cells also seen *in vivo* model

**Figure 7:**
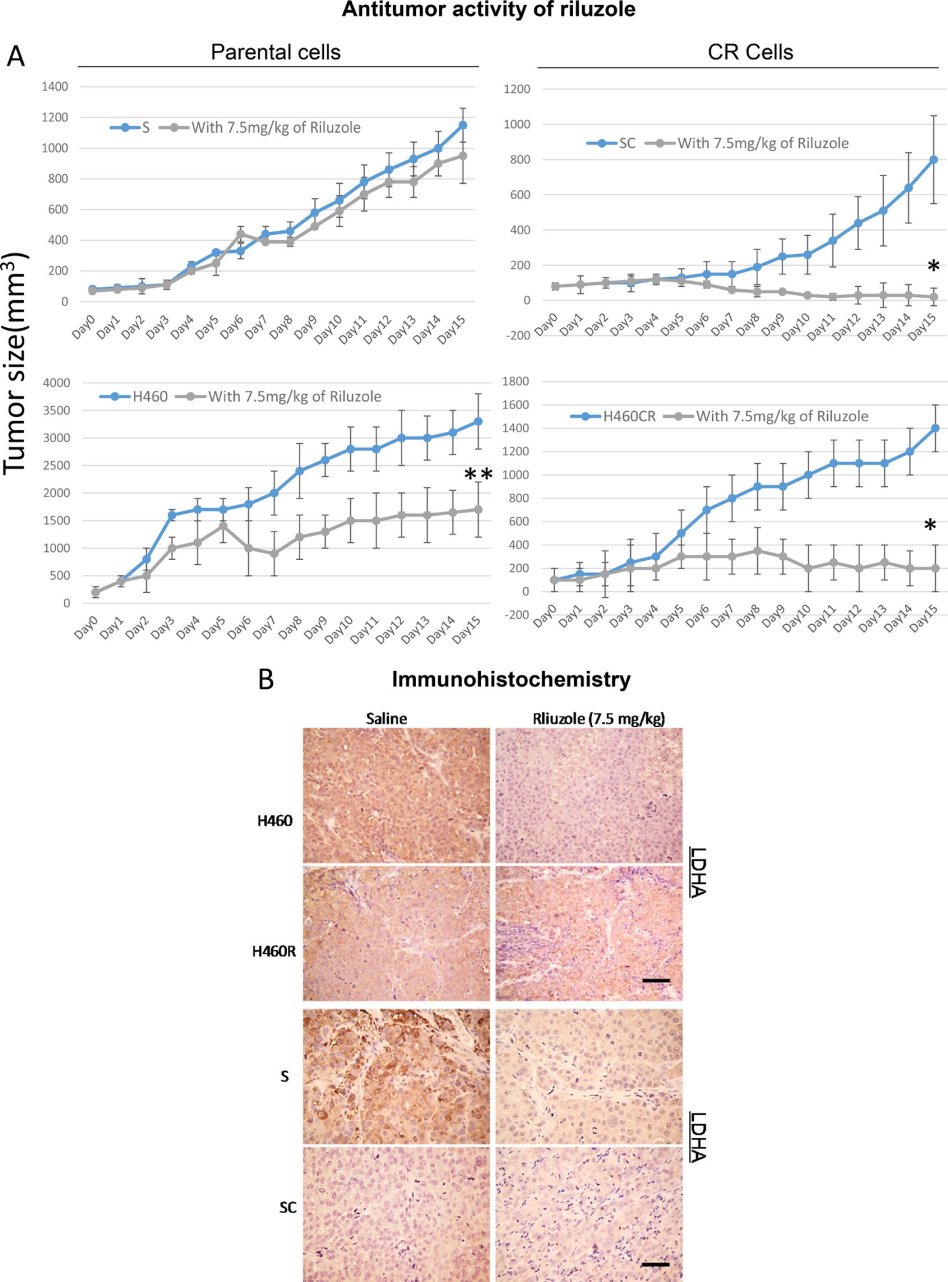
Riluzole exerts antitumor activities in animal (**A**) *In-vivo* antitumor activity of riluzole in parental vs. CR cells xenografts. Tumor growth was significantly reduced in the CR tumor groups (*P < 0.001). Noticeable tumor size reduction in H460 mice model was also observed (**P < 0.04). (**B**) The levels of LDHA were detected in S/SC and H460/H460CR xenograft tumors with and without riluzole treatment using IHC staining and visualized by light microscopy (scale bar = 100 μm).

To address that antitumor activity of riluzole which we observed *in vitro* can be seen *in vivo*, we tested riluzole in 2 pairs of CR lung cancer xenograft models using S vs. SC and H460 vs. H460CR. Tumors were subcutaneously injected and were allowed to grow to about 100 mm^2^ before treatment with saline or 7.5 mg/kg riluzole (once a day). Riluzole induced a significant reduction in tumor size in CR xenografts with greatest reduction in SC (Figure 7A). However, noticeable tumor size reductions were also seen in H460 but not in S xenograft model. These differences may be due to the fact that H460 harbored KRAS mutation and possess higher basal level of ROS while S which was a wild type and produced less ROS. These findings will need further investigation. H460 also possessed lower LDHA when compared to S cells, and importantly, lowered LDHA expression was observed in tumor tissue from SC and H460R xenograft treated with riluzole (Figure 7B). Together, our data suggested that riluzole was effective as a novel therapeutic modality for CR cells.

## DISCUSSION

Metabolic alterations are one of the hallmarks of cancer, controlling tumor progression and metastasis [5, 28]. Our previous work has shown that all CR lung cell lines possess higher basal levels of ROS when compared to their parental counterparts and normal cells. One of the major contributory factors to higher ROS is the reduction in thioredoxin-1 (TRX1) levels due to increased secretion [1]. The increased secretion can be detected in the media and in the serum from mice carrying CR xenograft. Studies have shown that over-expression of TRX1 increased HIF1α while transfection of non-functional TRX1 decreased HIF1α [29]. Since HIF1α also regulates enzymes involved in the glycolytic pathway, it is possible that decreased TRX1 and HIF1α protein expressions [1] found in CR cells also have a negative impact on glucose metabolism.

Although increased glucose metabolism in cancer cell has been hypothesized as the main carbon skeleton source of energy, we and other [30] have shown that CR cells are no longer addicted to the glycolytic pathway, but rather are dependent on amino acids for energy and biosynthesis. Key glycolytic enzymes HK2 and LDHA, as well as lactate production were decreased in all CR cell lines tested which corresponded to the resistance to glycolytic inhibitor, 2DG. We have also shown previously that SCLC-CR cells utilized fatty acids as their main energy source as evident by increased acetyl-CoA carboxylase (ACC) and fatty acid synthesis (FASN), the two key enzymes involved in the fatty acid synthesis. These cell lines also were hypersensitive to ACC and FASN inhibitors (TOFA and C75, respectively). Thus, in contrast to NSCLC-CR cells which rely more on the glutamine/glutamate metabolism pathway, SCLC-CR did not depend primarily on glutamine/glutamate axis.

Glutamine and glutamate flux have great impact on NSCLC-CR cells viability as shown here in this study. CR cells take up significantly greater amounts of glutamine and secreted higher levels of glutamate when compared to parental counterparts. There are reports which have shown that glutamine imported into cells is not totally utilized for anabolic metabolism [31]. Rather, a portion of glutamine/glutamate is shuttled out of the cell in exchange for amino acids that directly activate mTOR [32, 33]. Other amino acids such as arginine which can be converted to glutamate also were recently reported to play an important role in regulating mTOR activity [34]. Interestingly, we have previously reported that mTOR activities were higher in all CR cells and they were sensitive to mTOR inhibitors rapamycin or temsirolimus [35]. Hence, the uptake and export of glutamine/glutamate serves both as signal to mTOR and as a source of amino acids to promote protein translation and cell survival.

Increased glutamate efflux is also the important process for cells to generate glutathione (GSH) to cope with high intracellular ROS levels. This process is carried out through system xc^−^, a cystine-glutamate exchange transporter composed of xCT subunit [36]. xCT is a component of a plasma membrane transporter that mediates the cellular uptake of extracellular cystine in exchange for intracellular glutamate and plays a key role in GSH synthesis. The activity of xCT-mediated cystine uptake in cancer cells is known to be highly associated with tumor growth and chemo-resistance [36, 37]. CD44 together with xCT contributed to ROS defense by promoting the synthesis of reduced GSH [18, 19]. We showed here that higher basal levels of ROS found in all CR cells possessed higher expressions of xCT and CD44 when compared to parental counterparts. Thus, we suggested that glutamine is important to CR cell not only for OXMET, but also for maintaining oxidative balance. More importantly, treatment of riluzole disrupted the oxidative defense in CR cells by significantly reducing glutamate release which in turn suppressed GSH levels and is one of the mechanisms resulting in higher ROS accumulation (see Figure Diagram). However, we were intrigued by the fact that SSZ, an inhibitor of system xc^−^ pump, had very minimal effect on CR cells. Thus, other mechanisms must be involved in ROS generation by riluzole.

Decreased LDHA expressions found in CR cells could also contribute to higher basal levels of ROS since less lactate production may redirect more pyruvate into mitochondria. It is noteworthy that pyruvate can also be generated by mitochondria via malic enzyme. Thus, with low LDHA activity, pyruvate can be shuttle back into the mitochondria to replenish the TCA cycle. Heightened mitochondrial activity has long been associated with increasing ROS production. In fact, the largest contributor to cellular ROS is the mitochondria. In addition, NAD^+^ which is a crucial co-enzyme of all organisms' redox system can be categorized as “metabolic currency”. We presented the evidence that CR cells already operated at a very low metabolic currency rate. A key enzyme in NAD^+^ biogenesis, NMNAT2, was also significantly reduced in CR cells. Thus, further suppression in NAD^+^ will increase the deficit in the balance and consequently will lead to metabolic collapse. This is consistent with other reports which showed that certain cancer cells already have very low NAD^+^/NADH ration [38] and change in NAD^+^ correlates better with tumor growth than change in ATP [39]. Importantly, we found that riluzole can increase ROS by disrupting LDHA expression through suppression of NAD^+^. Reconstituted NAD^+^ re-stabilized LDHA which resulted in lowering intracellular ROS, and reversal of riluzole cytotoxicity. Note that NAD^+^ is a key factor for maintaining redox balance as it serves as a precursor to NADPH. Suppression of NAD^+^ most likely represents a new mechanism on how riluzole increased ROS levels in CR cells besides blocking xCT pump. Furthermore, NAD^+^ can stimulate PKA activity and in turn stabilize LDHA mRNA [25, 26]. Whether riluzole can effect PKA directly is not known, we plan to investigate this in the future.

Riluzole, an amino acid channel blocker, is taken orally and has generally been well tolerated by patients. Increasing ROS through riluzole pushes CR cells beyond their tolerance limit, which ultimately leads to cell death. In fact, recent report has shown that riluzole can increase ROS in tumor cells which further supports our findings [40]. Targeting cancer cells by ROS mediated mechanisms is an effective strategy to treat cancer cells that possess unique redox mechanisms. Consequently, it is possible to consider that riluzole may have dual-targeting action. Riluzole heightens ROS in CR cells by simultaneously suppressing LDHA and blocking xCT anti-port system. Thus, this could explain why SSZ alone, which only targets xCT, has a minimal effect in CR cells.

Moreover, it is noteworthy that one mechanism by which riluzole might be mediating its effect on lung cancer is by blocking ion channels, specifically tetrodotoxin–sensitive voltage-gated sodium channels (VGSCs) [41, 42]. It is well established that riluzole blocks the functions of these channels in neurons [41]. VGSCs have been shown to be expressed in a range of cell types including lung cancer cells [43]. However, the action of riluzole on these receptors, as well as the metabotropic receptors, has been controversial. In fact, there is no report on riluzole binding site to any of those receptors [44, 45].

In conclusion, we discovered that cisplatin resistant cells possess high levels of ROS and undergo metabolic reprogramming. These alterations can be exploited to kill CR cells by using riluzole. Riluzole treatment further increased ROS by multiple mechanisms. The key mechanism we found is that riluzole suppresses LDHA and NAD^+^ levels. Riluzole also blocks cystine/glutamate pump. Together, these two mechanisms work in conjunction to heighten ROS levels and lead to cell death in CR cells. Repurpose of riluzole as an antitumor agent against cisplatin resistance in lung cancer patients is possible and should be explored.

## MATERIALS AND METHODS

### Cell lines and reagents

Two pairs of cisplatin resistant from SCLC (SCLC1/ SR2 and H69/H69CR) and two pairs from NSCLC (H460/H460CR and S/SC) were used in this study. The characteristics of SCLC1/SR2 as well as S/SC have been previously characterized [35, 46–48]. H69, H460, and LL24 were purchased from ATCC. Their cisplatin resistant variants were established in our laboratory using previously published method [35, 47]. Note: SR2 exhibits 20 fold resistance, SC exhibits 7-fold resistance, while H69CR and H460CR exhibits 10-fold resistance to cisplatin. LL24 is normal lung fibroblast. Supplementary Table 1 showed the ID_50_ values for the sensitive and resistant cell lines. Riluzole was purchased from SelleckChem. NAC and NAD^+^were purchased from Sigma.

### Growth inhibition and cytotoxicity assay

Cells were seeded in 24-well dishes and treated with various concentrations of riluzole. The procedure was described previously [46, 49]. Briefly, the culture media as well as the trypsinized cells were collected and this mixture was centrifuged at 400 x *g* for 5 min. The supernatant was discarded, and resuspended in 1 mL of Hank's buffer and assayed for live cells and death cells using trypan blue exclusion method.

### Western blot analysis

Cells were seeded at 1 × 10^5^/ml onto 100 mm dishes, treated, collected, lysed and immunoblotted with indicated antibody. Detail procedure was described in our previous publications [46, 49]. Briefly, cell lysis was completed by sonication and the total protein was separated on an SDS-PAGE, transferred onto a PVDF membrane (Millipore) and immunoblotted with HK2, LDHA (Cell Signaling), xCT (BD Bioscience), CD44 (Santa Cruz), and Actin (Sigma). Bands were measured using a molecular imager Chemidoc system with Quality One software (Bio-Rad).

### Assay of Intracellular ROS/H_2_O_2_

As previously described [50], cells were collected and intracellular H_2_O_2_ was measured by incubating with either 10 μM of acetyl-penta-fluorobenzenesulfonyl fluorescein (APFB) (EMD) or 5 uM of MitoSOX (Invitrogen) at 37°C for 30 min in the dark. Then the cells were washed once with PBS and centrifuged to remove impermeable reagents. Cells were resuspended in 500 μL of PBS and analyzed in a fluorimeter, FLUOstar OPTIMA, BMG Labtech (excitation at 485 nm and emission at 520 nm).

### RNA interference experiments

8 × 10^5^ cells were seeded in a 60 mm petri dish and incubated for 24 h. INTERFERin^TM^ transfection reagent (Polyplus) was then used to transfect 1 nM of LDHA directed SMARTpool^®^ siRNA (NM_005566) or siCONTROL^®^ (5′-UAGCGACUAAACACAUCAA-3′) (Dharmacon). Cells were incubated in the presence of transfection medium for 24 h as previously described [49].

### Qualitative realtime PCR

qRTPCR was carried out as previously described [51]. Briefly, 1ug of RNA was used for cDNA synthesis. The primers for qRT-PCR are designed with Perlprimer for SYBR Green fluorophore. 40 cycle amplification was used. The data were analyzed with CFX manager software from Bio-Rad. To calculate the relative mRNA level, we used the ΔΔCt method. The level of mRNA was corrected with that of actin.

### Real time assay of oxygen consumption and lactate production

Simultaneous multi-parameter metabolic analysis of cell populations in culture was performed in the Seahorse XF^e^24 extracellular flux analyzer (Seahorse Bioscience) as described by Wu et al. [52]. All cell lines were cultured in growth medium for 24 h in plate before real-time metabolic analysis. At the start of the assay, growth medium was removed and replaced with assay medium. Basal OCR (oxygen consumption rate) and ECAR (extracellular acidification rate) of the cells were measured. After XF assay, cells were harvested by trypsin-EDTA treatment and counted. The number of cells per well were used to normalize OCR and ECAR.

### Animal studies

The procedures and protocol of mice were approved by the Institutional Animal Care and Use Committee (IACUC) of Miami VA medical center (Animal Welfare Assurance Number: A3739-01). Female athymic nude-Foxn1nu mice (6–8 weeks, Harlan Laboratories) were inoculated subcutaneously with 1 × 10^6^ cells prepared in physiologic buffered saline (PBS). When the tumor volumes reached 100 mm^3^, the treatment group received IP injection of riluzole (7.5 mg/kg) daily, and control group was saline only. The formula of tumor volume was (length × width^2^)/2. Tumor growth inhibition was calculated as median of tumor volume in treatment group to that in control group (T/C) ratio. NCI standard of the T/C ratio is < 42% which indicates significant tumor growth inhibition.

### Transmission electron microscopy (TEM)

Cancer cell cultures were acquired and immediately fixed in neutral buffered 2.5% glutaraldehyde at room temperature (25°C). The specimens were post-fixed in 1% osmium tetroxide (OsO_4_) for 10 minutes, dehydrated using a graded ethanol series, enbloch stained with 2% uranyl acetate in 50% ethanol for 30 minutes, and embedded in Spurr's epoxy resin. Semi-thin (1 μm) and ultra-thin (< 90 nm) sections were cut using a Diatome 3 mm diamond knife on the Leica EM UC6 ultramicrotome. Semi-thin sections were stained with toluidine blue and examined using an Olympus BX60 light microscope with an attached Olympus DP71 digital camera. All ultra-thin sections were stained using lead citrate to be viewed under TEM on a Jeol 1400 EM at 80 kV. Images for publication were compiled into using Photoshop CS5 software.

### Assay of mitochondrial membrane potential and activity (MMP)

Cells were collected and membrane potentials were measured by incubating with 50 nM of tetramethylrhodamine ethyl ester (TMRE) (Invitrogen) at 37°C for 30 min in the dark. Then the cells were washed once with PBS and centrifuged to remove impermeable reagents. Cells were resuspended in 500 μL of PBS and analyzed in FLUOstar OPTIMA (excitation at 544 nm and emission at 590 nm).

### Glutamine uptake

Cells were seeded and cultured for 24 h in complete RPMI media. The medium was then replaced with fresh “hot” (5μCi L-[G-^3^H] glutamine, PerkinElmer) glutamine free and serum free RPMI. Next, the hot medium was removed, cells were washed three times with fresh, RPMI serum-free with glutamine medium “cold”, and the cells were lysed with 0.5 mL of 1 N NaOH, collected in 1.5 mL Eppendorf tubes and gently vortexed. A 0.25 mL sample was used for protein analysis by Micro BCA Protein Assay Kit (Thermo Scientific). Radioactivity from the remaining sample was counted using a PerkinElmer Tri-Carb 2810TR liquid scintillation spectrometer with QuantaSmart. Radioactivity was measured as counts per minute (CPM) and normalized to protein.

### Glutamate release

The amount of glutamate that was transported out of the cells was measured using the Amplex^®^-Red Glutamic Acid/Glutamate Oxidase assay kit (Molecular Probes/Invitrogen). The assay is based on the oxidation of glutamate by glutamate oxidase, producing alpha-ketoglutarate, NH_3_, and H_2_O_2_. The H_2_O_2_ reacts with the Amplex^®^-Red reagent to produce the fluorescent product, risorufin. A standard curve from 0 to 20 μM was prepared using the kit's glutamate stock. The positive control was 10 μM H_2_O_2_ in reaction buffer. Cells were seeded in glutamine/glutamate free media and 50μL of media were then collected. 50 μL of Amplex^®^-Red working solution was added to each well, and the plate was incubated at 37°C for 30 min to 1 h. Fluorescence was detected at 590 nm using the OPTIMA plate reader. Concentrations of L-glutamate were calculated from the standard curve.

### Assay of glutathione (GSH)

A GSH assay kit (Cayman Chemical) was used to measure total cellular glutathione. Cells were seeded at 4 × 10^5^ and cell lysate was prepared by sonication and deproteination using the conditions recommended by the manufacturer. Total GSH was detected by measuring the product of glutathionylated DTNB by UV spectrophotometer at 405 nm. The cellular GSH contents were calculated using the standard curve generated in parallel experiments.

### Assay of nicotinamide adenine dinucleotide (NAD^+^)

A NAD/NADH assay kit (Cayman Chemical) was used to measure total cellular NAD^+^ concentration. Cell were seeded in 96-well at 1×10^4^ and grow overnight. Different dosages of riluzole were added on the following day for 1 h incubation period. Total NAD^+^ was detected at 450 nm according to manufacture instruction.

### Glucose uptake

To measure glucose uptake, fluorescently labeled glucose analogue was used. Cells were collected and incubated with 2-NBD glucose (Life Technologies) at 12.5 μg/ml for 30 minutes at room temperature. Cells were then washed twice with PBS and resuspended in 500 μL of PBS and analyzed in C6 Accuri Flow (excitation at 544 nm and emission at 590 nm).

### Immunohistochemistry staining

Tumor samples were immersed in 10% formalin for 2 week followed by 70% ethanol for a few days, and then dehydrated with gradient concentrations of ethanol and embedded in paraffin blocks. The tissue slides (4 μm) were dewaxed by xylene. Antigen retrieval was carried out with citric acid (10 mM, pH 6.0) containing 0.05% Tween 20. For IHC staining, the tumor tissue slides were hybridized with LDHA antibody (Cell Singling Technology, 1:400) at 4°C overnight. Thereafter, the slides were stained with LSAB™2 Kits (DAKO) and hematoxylin (DAKO) and visualized by a light microscope (Olympus).

### Amino acid analyzer

Free amino acids (30–40 in the complete panel) in physiological fluids were analyzed by Biochrom 30 amino acids analyzer using ion exchange chromatography. Values were reported compared to control normal ranges. Ion exchange chromatography is based on analytical separation with ion-exchange column and buffers of increasing pH and ionic strength. The chromatographically separated amino acids were detected after post-column derivatization with Ninhydrin and colorimetric intensity recorded at wavelength of 570 nm and 440 nm. Concentration of amino acid was calculated from comparing peak area of a particular amino acid to peak area of internal standard-AEC of known concentration and then multiply by its specific response factor from calibration. Proline and hydroxyl-proline were calculated from 440 nm, rest of AAs is from 570 nm.

### Statistical analysis

All statistical analyses were performed from three separate measurements using the two-tailed *t-test* and the results were expressed as mean ± standard deviation. A *p-value* of less than 0.05 was considered as statistically significant. As for TEM, mitochondrial area per cell area ratios between H460 and H460R were statistically analyzed using difference of means function from the InStat program. Comparisons were made using a two-tailed Paired *t-test*. The level of statistical for comparison was set at *p* = 0.05.

## SUPPLEMENTARY MATERIALS FIGURES AND TABLE



## Abbreviations

CR: cisplatin resistant
ROS: reactive oxygen species
OXMET: oxidative metabolism
OXPHOS: oxidative phosphorylation
HK2: hexokinase-2
LDHA: lactate dehydrogenase A
NAD^+^: nicotinamide adenine dinucleotide
GSH: glutathione
TRX1: thioredoxin-1
2-DG: 2 deoxy-D-glucose
SCLC: small cell lung cancer
NSCLC: non-small cell lung cancer
