# Supplementary material for: Exploiting ROS and metabolic differences to kill cisplatin resistant lung cancer
Source: Oncotarget. 2017 May 2;8(30):49275–92. doi: 10.18632/oncotarget.17568 (PMC5564767; doi:10.18632/oncotarget.17568)
Supplement: Supplementary file 1 [file oncotarget-08-49275-s001.pdf]

Exploiting ROS and metabolic differences to kill cisplatin resistant lung cancer

Supplementary Materials

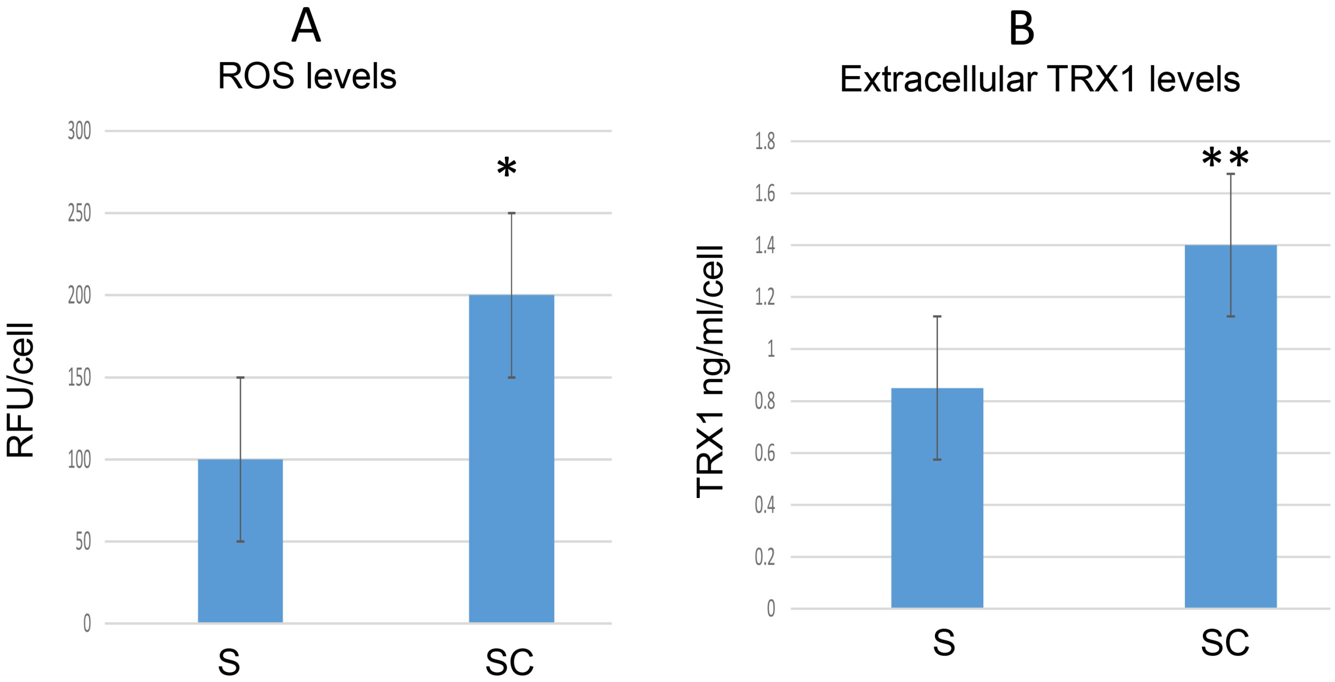

Supplementary Figure 1: CR lung cancer cells express higher ROS and secrete higher TRX1. (**A**) Fluorometer analysis of ROS detected by APFB probe indicated that CR lung cancer cell line expressed higher basal levels of ROS. Bar graph represents the relative fluorescent units/cell via fluorometer plate reader; (Mean SD of three experiments). (**B**) The concentration of extracellular TRX1 in culture medium. CR cells secreted greater levels of TRX1 when compared to their parental counterparts (**P* = 0.001, ***P* = 0.002).

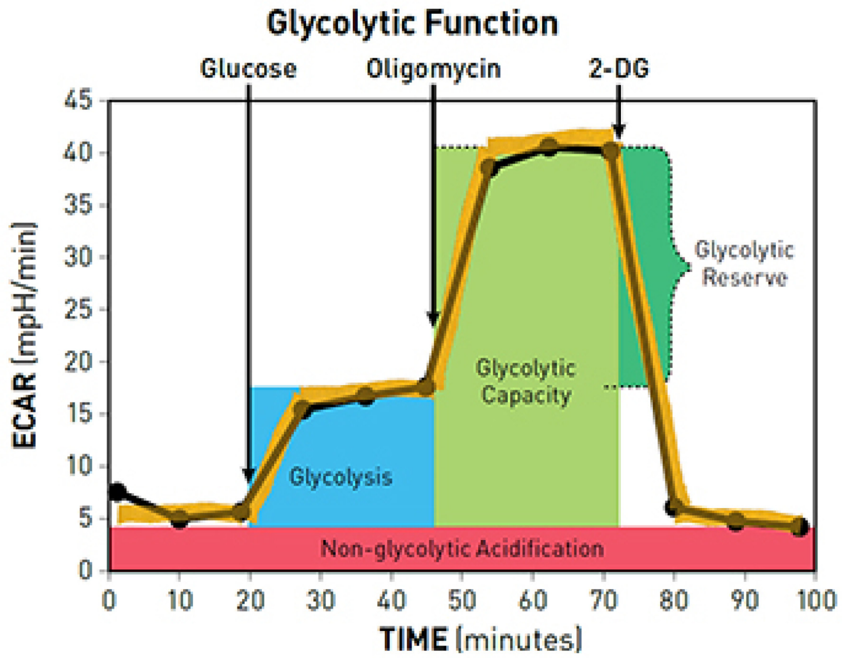

Supplementary Figure 1.1: Schematic of the Glycolytic Function test. ECAR (extracellular acid rate or lactate production) is measured by using different inhibitors. After glucose injection, oligomycin is injected to inhibit ATP synthase and shifts the energy production to glycolysis, with the subsequent increase in ECAR revealing the cellular maximum glycolytic capacity. The final injection is 2-DG which inhibits. The resulting decrease in ECAR confirms that the ECAR produced in the experiment is due to glycolysis.

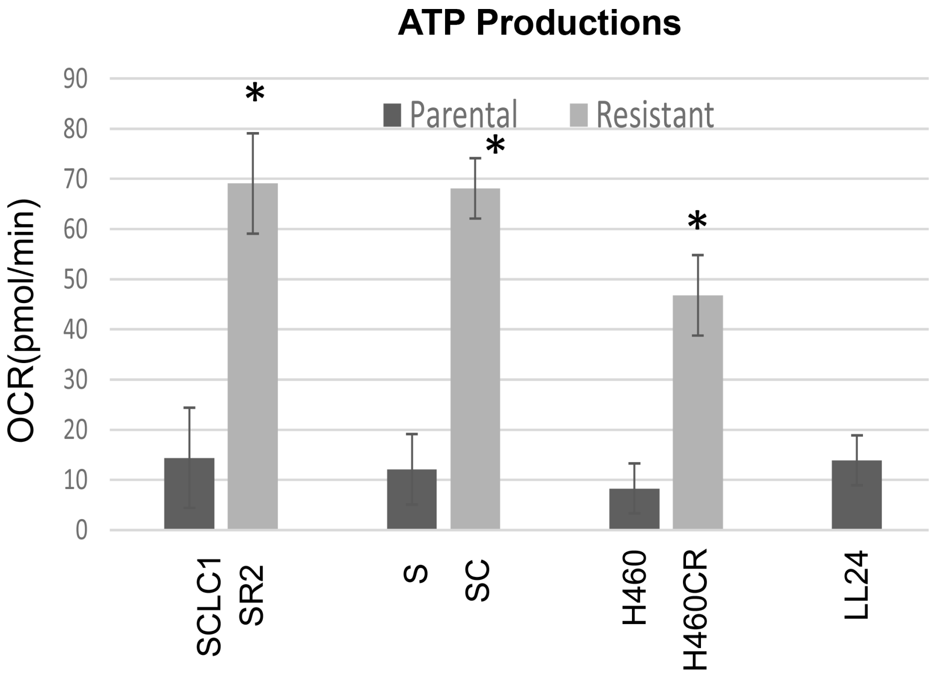

Supplementary Figure 2: ATP Productions. Parental and CR cells were assayed for baseline ATP production using Seahorse analyzer. CR cells possessed higher number of mitochondrial and therefore produced more ATP. (*$p < 0.03$).

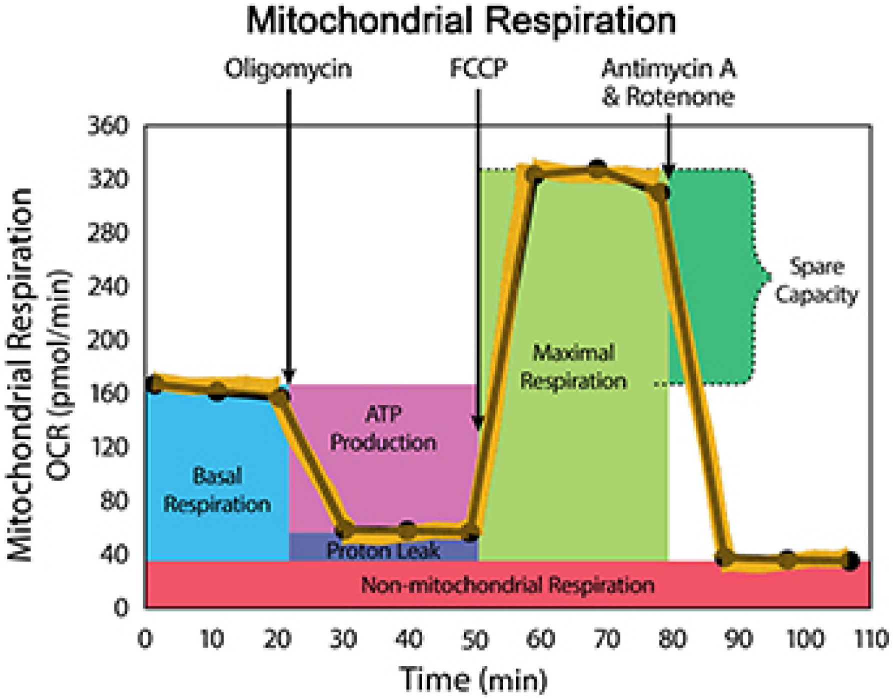

Supplementary Figure 2.1: Schematic of the mitochondrial Stress test. OCR (oxygen consumption rate) of mitochondrial respiration is measured by using different mitochondrial inhibitors and uncoupler. The basal level of oxygen consumption is measured before adding oligomycin, which represents the basal OCR. Oligomycin is injected to inhibit ATP synthase. To determine the maximal oxygen consumption rate, the uncoupler FCCP is injected. A mixture of rotenone/antimycin A is injected to inhibit the flux of electrons through complex I and III, and thus shutting down mitochondrial respiration.

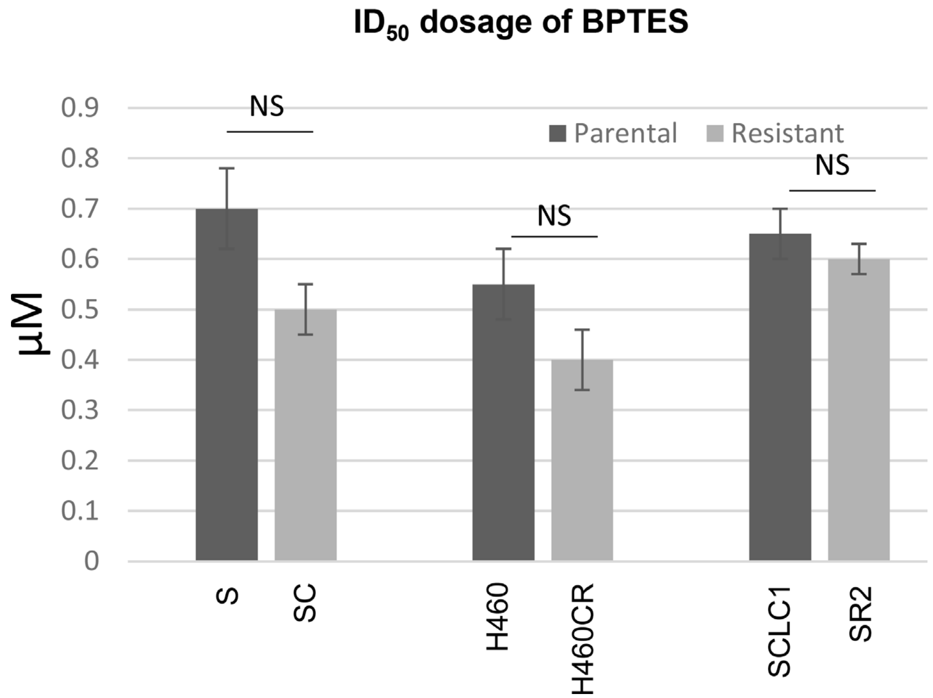

Supplementary Figure 3: Growth inhibitory dosage (ID50) of BPTES for 72 h showed that only NSCLC-CR (SC and H460CR) which possessed higher GLS expressions were more sensitive to riluzole. (Mean SD of three experiments)

A

ID_{50} dosage of riluzole

**SCLC1**	**15 ± 2.5**
**SR2**	**5 ± 1.2**
**S**	**14 ± 2.1**
**SC**	**2 ± 0.25**
**H460**	**5 ± 0.3**
**H460CR**	**3 ± 0.36**

B

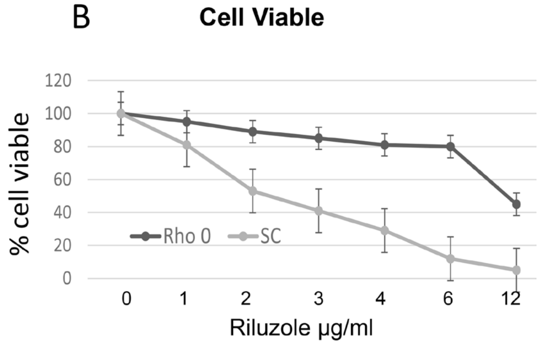

C

ID_{50} dosage of SSZ

	**ID_{50} µM**
**S**	**260± 10.2**
**SC**	**250± 8.5**
**H460**	**400± 12.6**
**H460R**	**420± 10.7**

Supplementary Figure 4: Riluzole induced CR cells death through mitochondria ROS. (**A**) Growth inhibitory effect of riluzole for 72 h in parental vs. CR cells. CR cells were sensitive to riluzole treatment. (**B**) Growth inhibitory effect of riluzole for 72 h. Rho 0 cells were resistant to riluzole treatment. (**C**) Growth inhibitory dosage (ID50) of sulfasalazine (SSZ) for 72 h. CR cells were not sensitive to SSZ (Mean SD of three experiments).

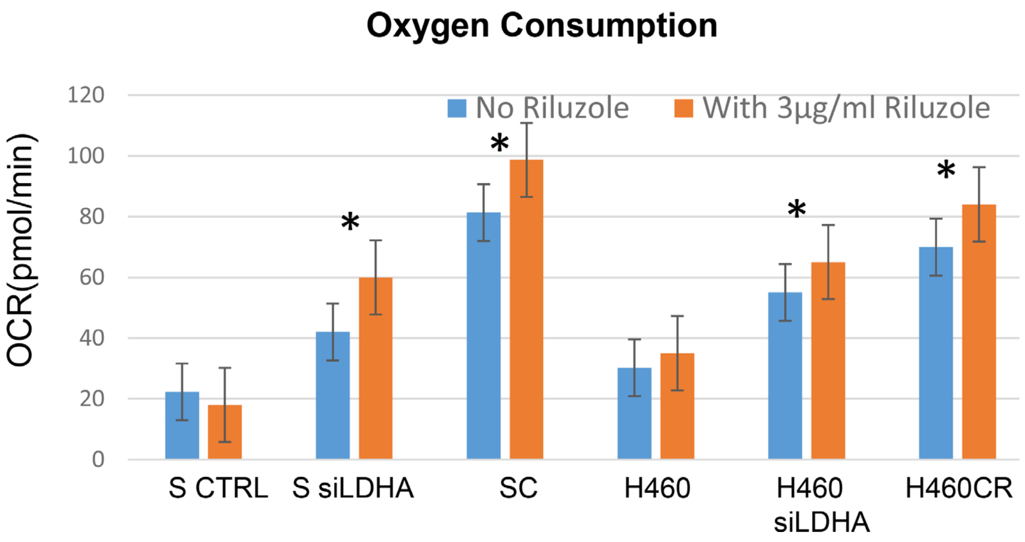

Supplementary Figure 5: Cells were assayed for oxygen consumption using Seahorse XFe24 extracellular flux analyzer. The rate of oxygen consumption (OCR) were higher in CR cells as well as in LDHA knocked-down cells, and further increased upon riluzole treatment (*$P < 0.05$).

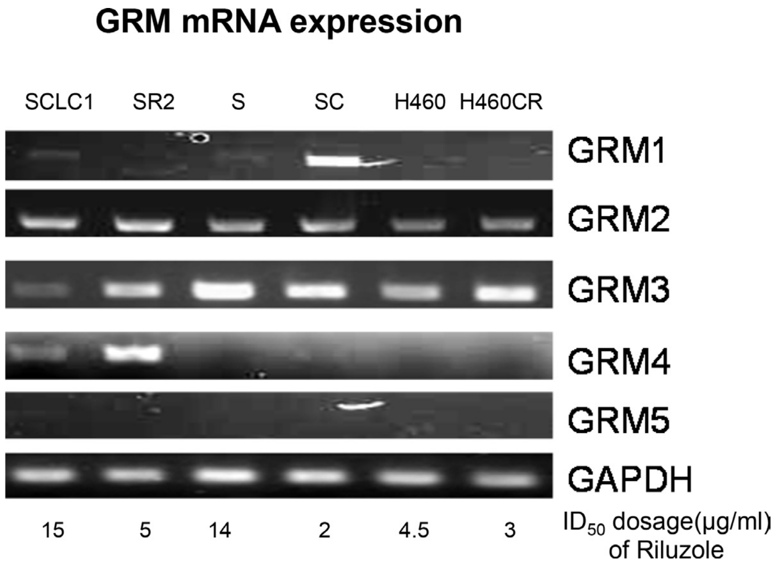

Supplementary Figure 6: mRNA expressions of GRM1-5 using PCR. GAPDH were used as control.

Supplementary Table 1: Growth inhibitory dosage (ID50) of cisplatin (72 h)

	SCLC				NSCLC			
	P	**CR**	***P***	**CR**	***P***	**CR**	***P***	**CR**
	SCLC1	SR2	H69	H69CR	S	SC	H460	H460CR
ID_{50} dosage (μg/ml)	0.15	2.5	0.1	1	0.3	3	0.15	1
Fold resistance		**16.7x**		**10x**		**10x**		**6.7x**

Note: P = parental cells and CR = cisplatin resistant cells.
